# Enhanced Upregulation of CRH mRNA Expression in the Nucleus Accumbens of Male Rats after a Second Injection of Methamphetamine Given Thirty Days Later

**DOI:** 10.1371/journal.pone.0084665

**Published:** 2014-01-27

**Authors:** Jean Lud Cadet, Christie Brannock, Bruce Ladenheim, Michael T. McCoy, Irina N. Krasnova, Elin Lehrmann, Kevin G. Becker, Subramaniam Jayanthi

**Affiliations:** 1 Molecular Neuropsychiatry Research Branch, Intramural Research Program, National Institute on Drug Abuse, National Institutes of Health, Baltimore, Maryland, United States of America; 2 Gene Expression and Genomics Unit, Intramural Research Program, National Institute on Aging, National Institutes of Health, Baltimore, Maryland, United States of America; Max Planck Institute of Psychiatry, Germany

## Abstract

Methamphetamine (METH) is a widely abused amphetamine analog. Few studies have investigated the molecular effects of METH exposure in adult animals. Herein, we determined the consequences of an injection of METH (10 mg/kg) on transcriptional effects of a second METH (2.5 mg/kg) injection given one month later. We thus measured gene expression by microarray analyses in the nucleus accumbens (NAc) of 4 groups of rats euthanized 2 hours after the second injection: saline-pretreated followed by saline-challenged (SS) or METH-challenged (SM); and METH-pretreated followed by saline-challenged (MS) or METH-challenged (MM). Microarray analyses revealed that METH (2.5 mg/kg) produced acute changes (1.8-fold; *P*<0.01) in the expression of 412 (352 upregulated, 60 down-regulated) transcripts including cocaine and amphetamine regulated transcript, corticotropin-releasing hormone (*Crh*), oxytocin (*Oxt*), and vasopressin (*Avp*) that were upregulated. Injection of METH (10 mg/kg) altered the expression of 503 (338 upregulated, 165 down-regulated) transcripts measured one month later (MS group). These genes also included *Cart* and *Crh*. The MM group showed altered expression of 766 (565 upregulated, 201 down-regulated) transcripts including *Avp*, *Cart*, and *Crh*. The METH-induced increased *Crh* expression was enhanced in the MM group in comparison to SM and MS groups. Quantitative PCR confirmed the METH-induced changes in mRNA levels. Therefore, a single injection of METH produced long-lasting changes in gene expression in the rodent NAc. The long-term increases in *Crh*, *Cart*, and *Avp* mRNA expression suggest that METH exposure produced prolonged activation of the endogenous stress system. The METH-induced changes in oxytocin expression also suggest the possibility that this neuropeptide might play a significant role in the neuroplastic and affiliative effects of this drug.

## Introduction

Methamphetamine (METH) is an indirect agonist that induces the release of dopamine (DA) in brain regions that receive projections from the substantia nigra pars compacta and the ventral tegmental area [1]–[3]. These brain regions include the nucleus accumbens and the dorsal striatum. METH administration also influences striatal gene expression in animals with normal dopaminergic innervation [4]–[6]. The METH-induced transcriptional changes in the dorsal striatum include increases in the expression of various immediate early genes (IEGs) including *c-fos* and *Egr* families of transcription factors, neuropeptides including neurotensin, and genes that participate in either toxic or protective cascades such as heat shock proteins and genes involved in endoplasmic reticulum stress, depending on the doses of METH used [5]–[9]. We have shown, in addition, that some of these changes can be attenuated by DA receptor antagonism [6]–[8] or repeated METH injections [5]. Nevertheless, these studies had only included very short-term biochemical or transcriptional effects of the drug and focused mainly on the dorsal striatum. Moreover, although the acute changes in METH-induced gene expression [7] and the toxic effects of the drug [6], [8], [10] have been extensively investigated in the dorsal striatum, very few papers have reported on the potential long-term behavioral and/or biochemical effects of a single injection of moderate doses of the drug. For example, Xi et al. [3] have shown that a single METH (10 or 20 mg/kg) injection can increase cocaine self-administration measured several days after the METH injection, thus documenting long-term behavioral effects of the drug. They showed that these METH doses also impacted the biochemical effects of cocaine in the nucleus accumbens [3]. More recently, Martin et al. [11] investigated the biochemical and molecular effects of a single METH (20 mg/kg) injection and identified substantial time-dependent changes in gene expression, histone acetylation, and expression of histone deacetylases (HDACs) in the NAc. We are, however, not aware of any study that has investigated the molecular effects of re-exposing rats to METH after a long period of abstinence following the injection of a single moderate but nonlethal dose of the drug. Moreover, to our knowledge, there is no study of the long-term effects of single or multiple exposures to the drug on global gene expression in the rat NAc, given the importance of that structure in reward mechanisms [12], [13].

Repeated injections of psychostimulant are the most often used model to examine the long-term effects of these drugs [14]. These studies have reported substantial activation of the mesolimbic dopaminergic projections [15]. However, there is evidence that even a single dose exposure can cause long-term alterations in dopaminergic systems, neuroendocrine, and physiological effects in rodents [16]–[19]. Specifically, Peris and Zahniser [17] showed that a single injection of cocaine caused potentiation of amphetamine-induced DA release from rat striatal slices. In rats, a single prior cocaine injection augmented a second cocaine injection-induced striatal DA release measured one week later [16]. Vanderschuren et al. [19] showed that the injection of a larger dose of amphetamine (5 mg/kg) injection also potentiated the biochemical effects of the injection of a second smaller dose of amphetamine (1 mg/kg) given 3 weeks later. Thus, when taken together with the behavioral and biochemical effects reported after a single METH pre-exposure [3], the possibility existed that a single METH injection might cause long-term biochemical and molecular changes in the rat NAc. We also tested the idea that such a moderate dose of METH might potentiate the molecular effects of the injection of a second lower dose of the drug in a fashion previously reported after a similar pattern of amphetamine injections [19]. In order to address these questions further, we used a two-dose METH exposure paradigm similar to that used by Vanderschuren et al. [19] to measure the effects of METH on gene expression in the NAc by using both microarray and quantitative PCR analyses. Thus, the purpose of the present paper was three fold. First, we sought to determine the acute effects of a single METH dose (2.5 mg/kg) on global gene expression in the NAc. We have previously shown that similar doses of METH can cause substantial changes in gene expression in the dorsal striatum [4], [20] but, to our knowledge, there are no similar data on the effects of similar doses of METH on global gene expression in the NAc. The second purpose of the study was to investigate the long-term effects of a moderate METH dose (10 mg/kg) in that brain structure. The studies that have investigated the effects of larger doses of METH (20–40 mg/kg) have reported on relatively short-term transcriptional effects of the drug on the cortex [6], [9], [21]. This issue is also important because we have shown that a single moderate dose of the drug can have long-term behavioral and biochemical effects [3], [22], results that suggest the possibility of long-lasting transcriptional effects of the drug. The third aim of the paper was to test if the single moderate dose of the drug could influence the transcriptional effects of a lower dose of the drug given one month later. We and others have reported that repeated injections of METH can attenuate the IEG [5], [20], toxic [23], and biochemical [24] responses to either smaller or larger doses given within a few hours after the end of the repeated METH injections. Frankel et al. [25] had also reported that a prior injection of a larger METH dose caused a potentiated locomotor response to a lower dose of the drug. Taken together, the literature suggests prior exposure to METH can influence subsequent exposure to a lower dose of the drug. However, we are not aware of any studies that have measured acute METH-induced changes in gene expression in the NAc after a long delay from an initial METH exposure. The present study was meant, in part, to fill that gap. These types of studies might be relevant to the effects of the drug on the brains of patients who go back to using drugs after long periods of abstinence.

In addition to measuring global gene expression, we used Ingenuity Pathway Analysis (IPA) to identify networks and canonical pathways that might be perturbed after injections of the drug. Our study reveals that a moderate dose of METH (10 mg/kg) can cause long-lasting changes in the mRNA expression of several neuropeptides including CRF, CART, AVP, and OXT in the NAc. Moreover, we showed that a prior exposure to METH (10 mg/kg) significantly influenced the acute transcriptional effects of a second delayed smaller dose of the drug (2.5 mg/kg) injection. These results are discussed in view of their support for the potential involvement of these neuropeptides in the psychostimulant-induced molecular neuroadaptations in the NAc.

## Results

### Monoamine levels in the NAc

In order to investigate the effects of METH pretreatment, we performed HPLC analyses in four experimental groups: saline-pretreated and saline-challenged (SS) (n = 4); saline-pretreated and METH-challenged (SM) (n = 8); METH-pretreated and saline-challenged (MS) (n = 9); and METH-pretreated and METH-challenged (MM) (n = 9). Table 1 shows the effects of METH on monoamine levels in the NAc of these rats. There were no significant differences in DA and 3, 4-dihydroxyphenylacetic acid (DOPAC) between the SS and MS groups. There were non-significant increases (+63%, *P* = 0.076) in homovanillic acid (HVA) levels in the MS in comparison to the SS group. The acute METH injection caused significant increases in DA and HVA levels in the saline- (SM) (+31.6% and +75%, respectively) and METH-pretreated (MM) (+40.8% and +98%, respectively) groups in comparison to the SS group. In addition, DA levels were significantly higher in the MM (+24.5%) in comparison to the MS group. There were no significant differences in DA, DOPAC, or HVA levels between the SM and MM groups. Serotonin (5-HT) and 5-hydroxyindole acetic acid (5-HIAA) levels were not significantly affected by any of the METH treatments.

**Table 1:** Effects of METH on monoamine levels in the NAc.

Amines	SS	SM	MS	MM
DA	6.67+0.95	8.78+0.51^a^	7.54+0.90	9.39+0.55^b^^,^ c
DOPAC	1.27+0.19	1.37+0.07	1.44+0.10	1.54+0.55
HVA	0.58+0.17	1.02+0.11^a^	0.94+0.10	1.15+0.13^b^
5-HT	1.18+0.13	1.20+0.11	1.17+0.10	1.16+0.08
5-HIAA	0.84+0.08	0.95+0.08	0.90+0.09	0.97+0.08

The values represent means + SEM (ng/mg tissue) per group saline-pretreated and saline-challenged (SS) (n = 4); saline-pretreated and METH-challenged (SM) (n = 8); METH-pretreated and saline-challenged (MS) (n = 9); and METH-pretreated and METH-challenged (MM) (n = 9).

a p<0.05;

b p<0.01 in comparison to the SS group;

c p<0.05 in comparison to the MS group.

No significant differences were observed between the SM and MM groups.

### Microarray analyses in the NAc

**Figure 1:**
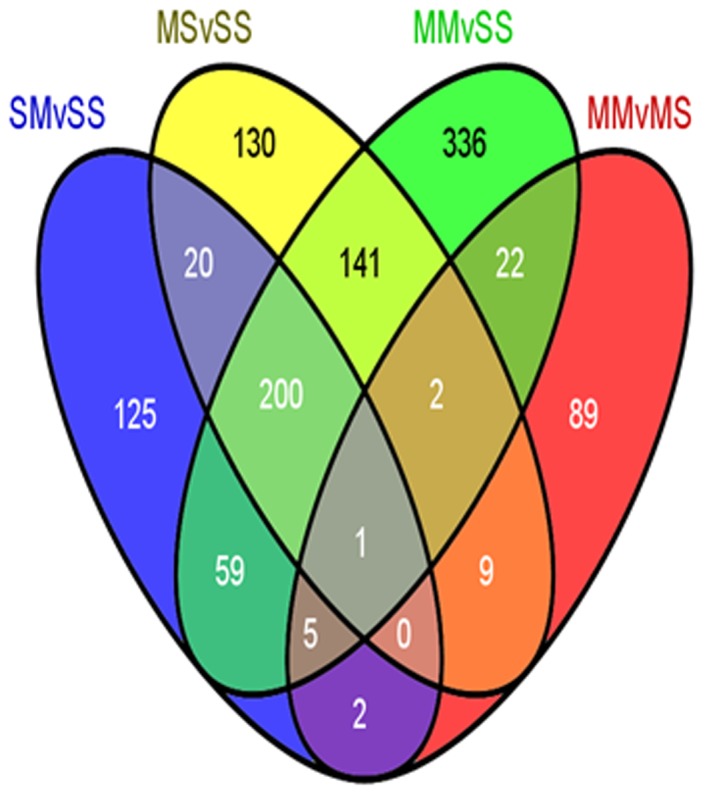
A single injection of METH (10 mg/kg) caused long-lasting changes in gene expression in the rat nucleus accumbens. The Venn diagram shows the overlap of genes in the four comparisons described in the text: SMvSS, MSvSS, MMvSS, and MMvMS. The rats were treated as described in the text and the animals were euthanized 2 hours after the second injection of either saline or METH. The microarray experiments were performed as described in the method section. Genes were identified as differentially expressed if they showed greater than+1.8-fold changes at *P*<0.01, using the GeneSpring statistical package.

In order to identify genes that are different between the four experimental groups (4 rats in SS; 6 rats in SM; and 7 rats in each MS and MM groups, see Table S1 in File S1), we performed microarray analyses using Rat Illumina arrays that contain 22,523 probes. The microarray data have been deposited in the NCBI database: GEO accession number GSE46717. We used a cut-off of 1.8-fold changes at *P*<0.01 because we have been able to replicate the changes in transcript levels by quantitative PCR analysis after identifying genes with similar criteria [5], [6], [11]. Figure 1 is a Venn diagram showing the effects of METH in four sets of comparison. Injection of METH (2.5 mg/kg) caused differential changes in the expression of a total of 412 transcripts (352 up-, 60 down-regulated) (SMvSS comparison). Injection of the larger METH (10 mg/kg) dose caused changes in the expression of 503 (338 upregulated, 165 down-regulated) transcripts in rats euthanized one month later (MSvSS comparison). Injection of METH (2.5 mg/kg) caused significant changes in 766 (565 upregulated, 201 down-regulated) transcripts in animals previously treated with a METH (10 mg/kg) injection one month earlier (MMvSS comparison). The single METH injection altered the expression of 130 transcripts (89 upregulated, 41 down-regulated) in animals previously treated with the METH (10 mg/kg) one month previously when compared to METH-pretreated rats challenged with saline (MMvMS comparison). There was a substantial degree of overlap in the identity of genes differentially expressed in the SMvSS and MSvSS comparisons, with 221 genes coexisting between these two comparisons. There were 344 genes located in the overlap between the MSvSS and the MMvSS comparisons while 265 genes were found in the overlap between the SMvSS and MMvSS comparisons. Interestingly, 201 genes were found in the SMvSS, MSvSS, and MMvSS comparisons, suggesting that the expression of many genes affected acutely by METH remained significantly altered for a period of, at least, one month after the injection.

**Figure 2:**
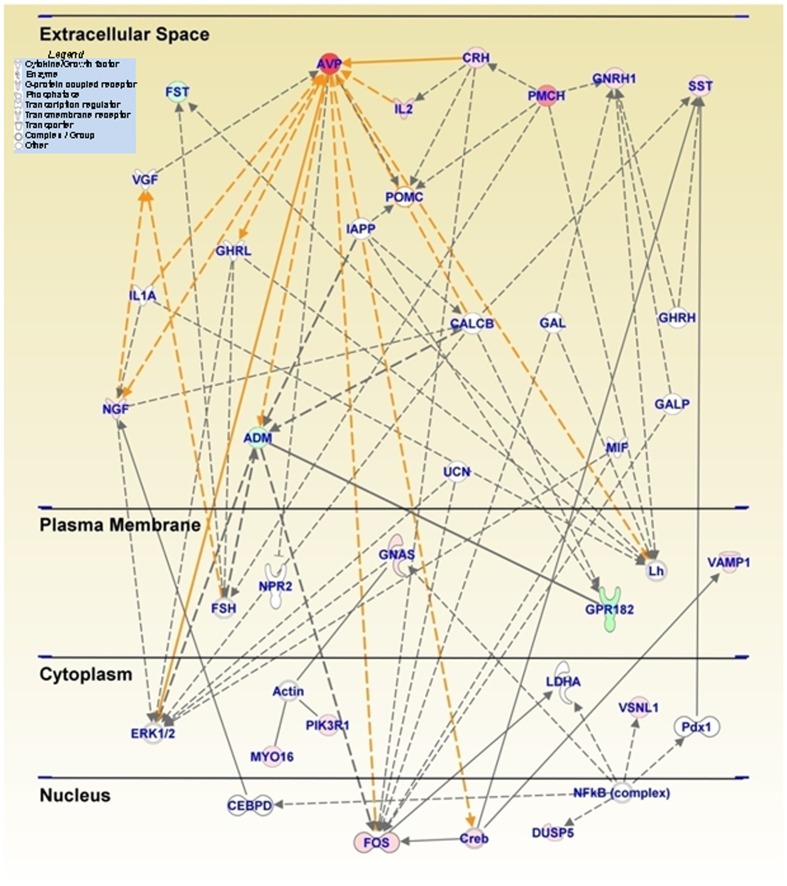
An acute METH injection induces changes in a network of genes that participate in cell and CRF signaling. The networks of related genes were generated through the use of IPA (Ingenuity® Systems, www.ingenuity.com). This figure shows that the relationship of several neuropeptides including Avp, Crh (Crf), and Sst that were significantly induced after the acute METH (2.5 mg/kg) injection. The genes were a subset of genes from the SMvSS comparison shown in figure 1. Relationships are shown as lines and arrows. The genes colored red to pink are up-regulated whereas those colored deep to light green are down-regulated. The intensity of the color represents is proportional to fold changes. The indirect relations between the genes were shown in dotted arrows and direct interaction in solid arrows. Arrows are colored differently to ease the identification of each connection. The various shapes within the figure represent the functional classes of the specific gene products (see legend in the top left).

Table 2 shows a partial list and the classes of genes that are up-regulated in comparison. to the SS group. Genes with increased expression in SMvSS include *Avp* (∼26.5-fold); *Cart* (7.6-fold); *Nr4a3* (5.85-fold); *c-fos* (5.25-fold); *Crf/Crh* (5.06-fold) and *Sst* (1.87-fold). The abbreviations are listed in the table. The METH-induced changes in immediate early genes (IEGs) are consistent with our previous observations that single or multiple injections of the drug can cause significant increases in striatal IEG expression [4]–[6], [8]. Pathway analysis using the IPA program identified several networks in which the METH-regulated genes participate. These include cell signaling, cell-to-cell signaling and interaction, nervous system development, and endocrine system functions. Top canonical pathways include G-Protein-coupled receptor signaling. Figure 2 shows a network that contains genes that are involved in cell signaling, CRH signaling and other endocrine functions. The activation of these endocrine signaling pathways after METH supports the suggestions that various peptide neurotransmitters might be involved in both the acute and long-term effects of drugs of abuse [26], [27] see discussion below). Figure 2 also provides evidence of METH induced regulation of genes connected to transcription regulation. These observations are consistent with those reported by several groups of investigators who had performed microarray analyses in forebrain dopaminergic projection areas of rodent brains [7]–[9], [11], [28]–[31].

**Table 2:** Partial list of METH-upregulated genes in comparison to SS group.

Symbol	Definition	Fold changes		
		SMvSS	MSvSS	MMvSS
*ADP-ribosylation*				
Art5	ADP-ribosyltransferase 5	2.36	**4.15**	**3.31**
*Axon guidance/cell cycle*				
Clasp1	cytoplasmic linker associated protein 1	2.25	3.09	**3.15**
Plxna4	plexin A4	**2.57**	1.15	2.00
*Cell adhesion*				
Cpne4	copine IV	5.48	**5.88**	8.48
Lypd3	Ly6/Plaur domain containing 3	3.85	**5.66**	**6.63**
Mpeg1	macrophage expressed gene 1	**5.52**	**4.27**	4.33
Parvb	parvin, beta	2.28	2.41	**3.14**
Pcdh18	protocadherin 18	4.99	**5.92**	7.16
Shank2	SH3/ankyrin domain gene 2	1.02	1.38	**3.54**
*Cell death*				
Gpx3	glutathione peroxidase 3	**2.13**	2.57	2.68
Nell1	NEL-like 1 (chicken)	3.90	**3.99**	**4.18**
Unc5d	unc-5 homolog D (*C. elegans*)	3.81	**5.36**	5.02
*Cell growth*				
Fgf11	fibroblast growth factor 11	1.30	2.73	**3.55**
Igfbp2	insulin-like growth factor binding protein 2	1.72	2.17	**4.94**
*Cell morphogenesis*				
Gbx2	gastrulation brain homeobox 2	5.07	**18.00**	20.82
Tnnt2	troponin T2, cardiac	**5.14**	**4.68**	4.94
Vax1	ventral anterior homeobox containing gene 1	2.41	**3.06**	2.57
*Defense/Immune systems*				
Calcr	calcitonin receptor,	**14.75**	**23.86**	**19.14**
Ccr1	chemokine (C-C motif) receptor 1	5.27	**4.18**	4.77
Cxcl13	chemokine (C-X-C motif) ligand 13	8.57	**11.49**	**12.93**
Defb1	defensin beta 1	6.62	**8.56**	**11.20**
Il2	interleukin 2	**7.02**	**4.67**	**5.03**
*Development*				
Aard	alanine and arginine rich domain containing protein	6.87	**8.40**	9.50
Chrd	chordin	3.46	**3.83**	2.72
Dlk1	delta-like 1 homolog (*Drosophila*)	6.87	**6.00**	5.74
Myh6	myosin, heavy polypeptide 6, cardiac muscle, alpha	2.42	2.81	**3.16**
Otx2	orthodenticle homolog 2 (*Drosophila*)	4.77	**4.75**	6.28
Rtbnd	retbindin	5.91	**6.30**	**4.93**
Susd3	sushi domain containing 3	3.90	**5.91**	3.61
*Homeostasis*				
Pmch	pro-melanin-concentrating hormone	15.83	**19.36**	**28.83**
Rxfp3	relaxin family peptide receptor 3	9.78	**9.32**	13.14
Tfrc	transferrin receptor	4.80	5.28	**5.55**
Agtr1a	angiotensin II receptor, type 1	2.17	**5.19**	6.66
Cckar	cholecystokinin A receptor	3.95	**6.58**	8.91
Cidea	cell death-inducing DNA fragmentation factor	1.65	**4.40**	3.10
*Intracellular protein transport*				
Rpl30	ribosomal protein L30	4.34	**5.64**	**7.18**
Slc7a3	solute carrier family 7, member 3	3.58	**3.90**	3.90
Stx17	syntaxin 17	**5.81**	3.55	5.81
Tmem7	transmembrane protein 7	**3.65**	**4.05**	**3.26**
Vamp1	vesicle-associated membrane protein 1	**2.18**	2.03	2.50
*Ion transport*				
Cacna2d2	calcium channel, voltage-dependent	21.16	10.84	**3.59**
Clcn1	chloride channel 1	**1.90**	1.62	1.41
Fstl5	follistatin-like 5	3.28	3.32	**3.39**
Gabra1	gamma-aminobutyric acid A receptor, alpha 1	3.35	**3.02**	4.79
Kcnc2	potassium voltage gated channel	**2.11**	2.00	2.12
Kcnj16	potassium inwardly-rectifying channel	**8.07**	**9.00**	**12.98**
Kcns3	potassium voltage-gated channel, delayed-rectifier	**2.68**	2.63	2.69
*Metabolic process*				
Cdk10	cyclin-dependent kinase 10	**2.07**	1.78	1.75
Gdpd2	glycerophosphodiester phosphodiesterase domain	3.22	**4.77**	4.08
Ptpn18	protein tyrosine phosphatase, non-receptor type 18	**5.81**	3.30	4.28
*Neuropeptides/Hormone activity*				
Avp	arginine vasopressin	**26.47**	10.84	**15.99**
Avpr1a	arginine vasopressin receptor 1A	**8.57**	**8.12**	**9.27**
Cart	cocaine and amphetamine regulated transcript	**7.63**	**5.65**	**4.97**
Crh	corticotropin releasing hormone	**5.06**	**4.00**	**7.33**
Gast	gastrin	**5.46**	5.01	**4.88**
Gnrh1	gonadotropin-releasing hormone 1	**4.01**	**3.56**	**4.45**
Nts	neurotensin	**2.09**	1.68	1.83
Oxt	oxytocin	11.50	7.98	**14.91**
Sst	somatostatin	**1.87**	1.58	1.73
Sstr1	somatostatin receptor 1	**2.98**	**3.27**	**3.97**
*Regulation of neurotransmitter*				
Chat	choline acetyltransferase	1.71	2.44	**3.04**
*Regulation of nucleotide*				
Adcy7	adenylate cyclase 7	2.54	**4.12**	**4.82**
Adcy8	adenylate cyclase 8	**2.30**	2.33	2.35
*Sensory perception*				
Otog	otogelin	**4.43**	1.22	1.79
*Signal transduction*				
Arc	activity regulated cytoskeletal-associated protein	**2.17**	-1.30	1.66
Calb2	calbindin 2	5.41	**6.09**	**6.01**
Camk1g	calcium/calmodulin-dependent protein kinase I gamma	**2.46**	1.91	2.48
Camk2d	calcium/calmodulin-dependent protein kinase II, delta	**2.71**	2.15	2.36
Cyp26a1	cytochrome P450, family 26, subfamily a, polypeptide 1	4.21	2.70	**3.71**
Disp2	dispatched homolog 2	2.64	3.38	**3.42**
Dusp5	dual specificity phosphatase 5	**2.43**	1.05	1.63
Gdap1l1	ganglioside-induced differentiation-associated protein 1	**1.84**	1.72	1.62
Gpr103	G protein-coupled receptor 103	**20.41**	**24.36**	**29.26**
Hap1	huntingtin-associated protein 1	**3.33**	**3.29**	**4.40**
Hcrtr2	hypocretin receptor 2	2.75	2.70	**3.08**
Ifitm6	interferon induced transmembrane protein 6	**2.16**	1.60	1.48
Insr	insulin receptor	2.33	2.61	**5.03**
Klhl12	kelch-like 12 (*Drosophila*)	**2.30**	1.89	1.91
Myo16	myosin XVI	**3.19**	3.33	**3.22**
Nmbr	neuromedin B receptor	**4.44**	2.78	3.93
Nnat	neuronatin	**5.85**	**5.09**	**5.25**
Nrip3	nuclear receptor interacting protein 3	2.20	2.58	**3.11**
Peli1	pellino homolog 1 (*Drosophila*)	**28.74**	**7.31**	**19.77**
Pnoc	prepronociceptin	**2.05**	1.97	2.08
Pth2r	parathyroid hormone 2 receptor	7.78	**11.84**	13.31
Stap2	signal transducing adaptor family member 2	2.96	**3.38**	2.70
Slc17a6	solute carrier family 17, member 6	4.66	**5.87**	6.45
Htr7	5-hydroxytryptamine receptor 7	3.03	**3.26**	3.31
*Structural*				
Myom3	myomesin family, member 3	**7.83**	**4.18**	**6.13**
Nup133	nucleoporin 133	1.32	1.93	**3.56**
Cbln2	cerebellin 2 precursor protein	1.94	**3.56**	**4.50**
*Transcription*				
Crebl2	cAMP responsive element binding protein-like 2	**2.69**	1.69	2.08
Egr2	early growth response 2	**2.68**	1.12	1.60
Egr4	early growth response 4	**3.24**	1.04	1.93
Fos	FBJ murine osteosarcoma viral oncogene homolog	**5.25**	1.35	**5.27**
Hsf4	heat shock transcription factor 4	2.63	**3.16**	**3.25**
Junb	Jun-B oncogene	**2.88**	1.24	2.12
Nkx2-5	NK2 transcription factor related, locus 5	2.24	1.69	**3.52**
Npas4	neuronal PAS domain protein 4	4.96	1.44	**5.08**
Nr4a3	nuclear receptor subfamily 4, group A, member 3	**5.85**	1.38	**3.94**
*Urea cycle*				
Arg2	arginase 2	**2.31**	1.98	2.15

The animals were treated and microarray analyses were performed as described in the text. The number listed in bold under the representative columns (SMvSS, MSvSS, MMvSS) identify genes whose mRNA were significantly increased according to the following criteria: greater than +1.8-fold, p<0.01. In some cases, values that are greater than 1.8-fold are not in bold because they did not reach the p value cut-off for the microarray analysis.

**Figure 3:**
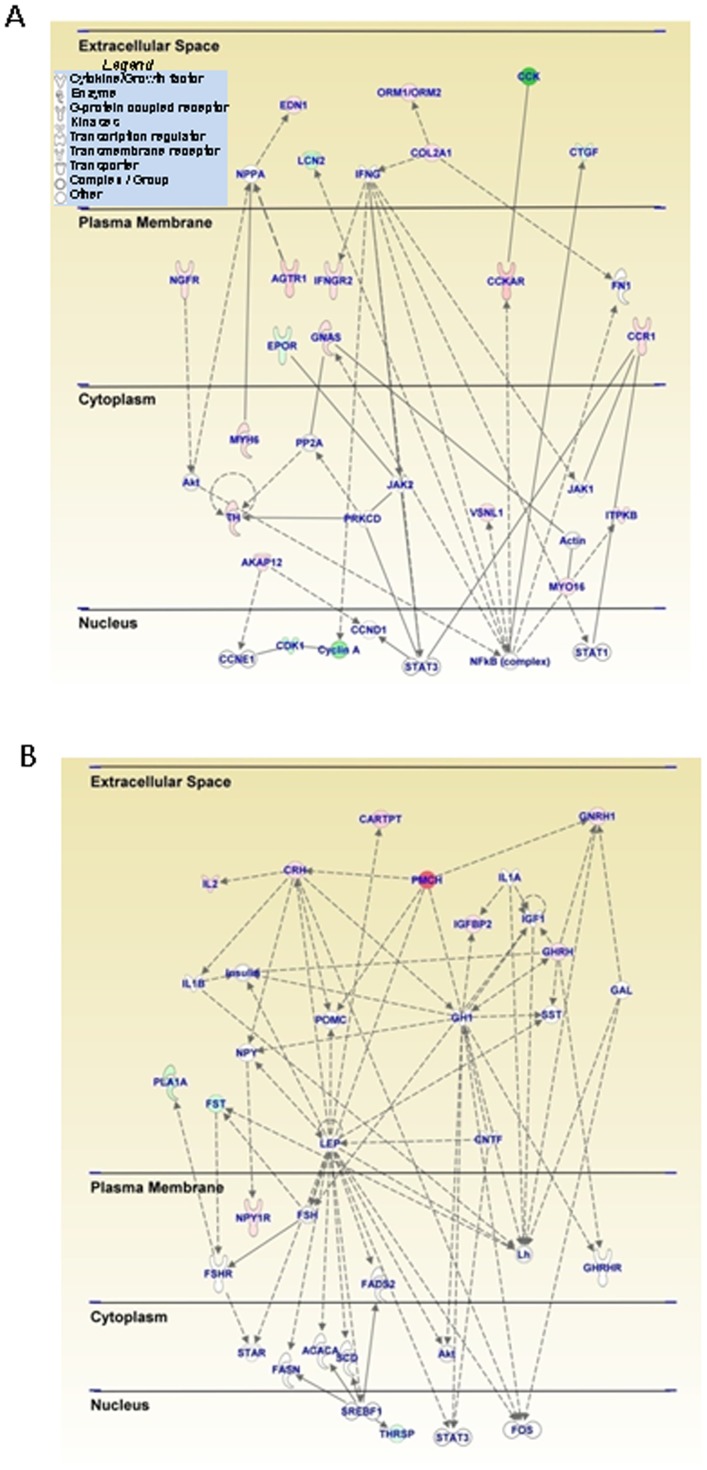
A METH (10 mg/kg) injection caused delayed changes in a network of genes involved in (A) cellular growth and proliferation and (B) in endocrine system regulation. This network of related genes was generated as described in figure 2. Relationships between genes are also described in figure 2. The rats were injected with METH (10 mg/kg) and were euthanized 2 hours after an injection of saline one month later. The genes were from the MSvSS comparison shown in figure 1. Several neuropeptides of interest including Cart and Crh (Crf) are upregulated one month after the single METH injection (**B**). In addition to the many upregulated genes, the figure 2A also shows that METH caused down-regulation of Cck mRNA one month after its injection.

Table 2 also shows a list of genes that were differentially expressed one month after a single injection of METH (10 mg/kg) (MSvSS comparison). The list includes *Cckar* (cholecystokinin A receptor) (6.58-fold), *Cart* (5.65-fold), *Crh* (4-fold), and *Gnrh1* (3.56-fold) that showed increased mRNA levels. Interestingly, there was METH-induced down-regulation of *Cck* (−3.43-fold) in that group (see Table 3 and Fig. 3A). Of interest is the fact that, in the MS group, there were no increases in the expression of any IEGs that were affected in the SM group. The present observations are consistent with those of a previous report that the induced IEG expression caused by METH (20 mg/kg) had reverted to normal by 16 hours after the drug injection [11]. IPA revealed that the genes whose expression was affected by the METH injection participated in cell death mechanisms, inflammatory responses, and endocrine functions. The activation of genes involved in death mechanisms and in inflammatory responses is consistent with previous data that have shown that METH can cause neurodegenerative changes [8], [10] and increased expression of neuroinflammatory markers [32], [33]. Canonical pathways of interest also included cAMP-mediated signaling, CRH signaling, and genes that are involved in the regulation of synaptic long-term potentiation. These observations are consistent with the fact that psychostimulant can cause prolonged changes in synaptic plasticity [34], [35]. IPA also identified networks of genes that are involved in cellular growth and proliferation (Fig. 3A) and in endocrine system disorders (Fig. 3B). The potential involvement of these genes and pathways in the acute and chronic effects of psychostimulant is discussed below (see also [27], [36]).

**Table 3:** Partial list of METH down-regulated genes in comparison to SS group.

Symbol	Definition	Fold changes		
		SMvSS	MSvSS	MMvSS
*Cell adhesion*				
Actn1	actinin, alpha 1	−1.65	−1.68	**−2.13**
Actn2	actinin alpha 2	−1.44	**−1.84**	**−2.05**
Ceacam10	carcinoembryonic antigen-related cell adhesion molecule 10	**−2.03**	−1.72	−1.95
Epor	erythropoietin receptor	−1.84	**−2.00**	−2.79
Ptpn7	protein tyrosine phosphatase, non-receptor type 7	−1.62	**−2.05**	**−2.14**
Ptprcap	protein tyrosine phosphatase, receptor type, C polypeptide-associated protein	−1.50	−2.47	**−3.53**
Ptprv	protein tyrosine phosphatase, receptor type, V	**−2.01**	**−2.12**	**−2.95**
Sema7a	semaphorin 7A, GPI membrane anchor	**−2.96**	**−3.16**	−2.77
*Cell cycle*				
Cdc20	cell division cycle 20	−1.31	−1.55	**−1.88**
Cdc2a	cell division cycle 2 homolog A (*S. pombe*)	−2.18	**−3.45**	**−4.68**
Cdca1	cell division cycle associated 1	−1.25	−1.61	**−1.93**
Cdca7	cell division cycle associated 7	−2.03	**−3.48**	**−5.46**
Cks2	CDC28 protein kinase regulatory subunit 2	−1.46	−2.20	**−2.58**
*Cell death*				
Bik	BCL2-interacting killer (apoptosis-inducing)	−1.85	−2.34	**−2.89**
Card6	caspase recruitment domain family, member 6	1.89	−1.27	**−2.45**
Lcn2	lipocalin 2	−2.58	**−3.08**	−2.06
Lyzl4	lysozyme-like 4	−1.62	**−2.19**	**−2.55**
Mzb1	marginal zone B and B1 cell-specific protein	−1.98	**−2.99**	**−2.48**
Osgin1	oxidative stress induced growth inhibitor 1	−4.59	−2.90	**−3.57**
Unc5b	unc-5 homolog B	−1.42	−1.37	**−1.90**
*Cell growth*				
Efemp2	EGF containing fibulin-like extracellular matrix protein 2	−1.51	**−1.83**	**−1.89**
Rhbdf1	rhomboid family 1	**2.27**	**−2.07**	−1.76
Tgfb1	transforming growth factor, beta 1	−1.84	**−1.85**	**−1.85**
*Cell organization*				
Acvr1c	activin A receptor, type IC	−2.26	**−2.58**	**−2.91**
Brsk2	BR serine/threonine kinase 2	−2.70	**−3.57**	**−3.44**
Cdc42ep1	CDC42 effector protein (Rho GTPase binding) 1	**−1.91**	−1.38	**−1.90**
Cenpe	centromere protein E	−2.95	−2.64	**−3.84**
Dscc1	defective in sister chromatid cohesion 1 homolog	−1.32	−1.60	**−2.45**
Kif11	kinesin family member 11	−2.70	−2.60	**−5.78**
Kif23	kinesin family member 23	−1.40	−1.73	**−2.29**
Kif4	kinesin family member 4	−3.70	**−7.04**	−3.43
Kifc1	kinesin family member C1	−2.34	**−1.93**	**2.02**
Mcm3	minichromosome maintenance complex component 3	−2.56	**−3.96**	**3.96**
Myh7b	myosin, heavy chain 7B, cardiac muscle, beta	−1.26	−1.94	**−2.93**
P2ry2	purinergic receptor P2Y, G-protein coupled, 2	**−3.05**	−1.95	−2.88
Pde10a	phosphodiesterase 10A	−1.55	**−2.03**	**−1.82**
Plk1	polo-like kinase 1 (*Drosophila*)	**−1.94**	**−2.70**	**−4.36**
Rsb66	Rsb-66 protein	−1.28	−1.27	**−2.15**
Hs3st2	heparan sulfate (glucosamine) 3-O-sulfotransferase 2	−1.58	**−2.56**	−2.16
*Cytoskeleton*				
Gypc	glycophorin C	−1.20	**−2.74**	**−2.75**
Krt17	keratin 17	**−2.15**	**−2.30**	**−2.86**
Obsl1	obscurin-like 1	−3.23	−2.62	**−4.32**
Synpo2	Synaptopodin 2	−1.33	**−2.13**	**−2.28**
Tnnt1	troponin T1, skeletal, slow	−1.52	**−2.42**	**−3.18**
*Defense/Immune response*				
Clcf1	cardiotrophin-like cytokine factor 1	−9.10	**−8.82**	−6.64
Cxcl11	chemokine (C-X-C motif) ligand 11	−4.22	**−4.21**	**−4.34**
Dmkn	dermokine	**−2.86**	**−2.86**	**−4.52**
Igsf9	immunoglobulin superfamily, member 9	−1.79	**−1.89**	**−2.33**
Il17re	interleukin 17 receptor E	−2.59	**−2.87**	−2.44
Irf6	interferon regulatory factor 6	**−3.06**	**−4.99**	**−4.63**
Itk	IL2-inducible T-cell kinase	−1.96	−1.79	**−2.91**
Mill1	MHC I like leukocyte 1	−1.25	−1.31	**−3.13**
Pik3cd	phosphatidylinositol-4,5-bisphosphate 3-kinase, catalytic subunit delta	**−2.30**	**−2.77**	**−2.76**
Pirb	paired-Ig-like receptor B	−1.50	**−2.90**	−1.96
Ptges	prostaglandin E synthase	**−3.19**	**−3.19**	**−2.91**
*Development*				
Adm	adrenomedullin	**−2.16**	−2.08	−1.30
Angpt2	angiopoietin 2	**−2.54**	**−3.03**	**−3.47**
Ascl1	achaete-scute complex homolog-like 1	**−1.64**	**−1.91**	**−2.13**
Cdr2	cerebellar degeneration-related 2	**−1.90**	**−2.44**	**−1.82**
Coch	cochlin	−1.81	**−2.23**	**−2.52**
Col18a1	procollagen, type XVIII, alpha 1	−1.77	**−2.13**	**−2.24**
Cryab	crystallin, alpha B	**−1.91**	**−2.21**	−1.69
Gfap	glial fibrillary acidic protein	**−2.75**	**−2.35**	**−2.22**
Plg	plasminogen	**−10.09**	**14.35**	**−16.06**
Slit3	slit homolog 3 (*Drosophila*)	**−1.93**	**−2.74**	**−2.68**
Zc3h12a	zinc finger CCCH type containing 12A	**−3.86**	−3.44	−1.68
*DNA repair*				
Ddit4l	DNA-damage-inducible transcript 4-like	−3.69	−4.39	**−5.63**
Mdc1	mediator of DNA-damage checkpoint 1	1.07	−1.01	**−1.89**
*Electron transport*				
Cyp4b1	cytochrome P450, family 4, subfamily b, polypeptide 1	−3.71	−2.68	**−3.25**
*Homeostasis*				
Ptchd2	patched domain containing 2	−1.84	**−2.00**	**−2.11**
*Ion binding*				
Arid5a	AT rich interactive domain 5A	−1.39	**−6.12**	1.23
Car7	carbonic anhydrase 7	**−2.04**	**−1.85**	**−2.04**
*Ion transport*				
Kcng1	potassium voltage-gated channel, subfamily G, member 1	−1.44	**−1.95**	−1.57
Kcnh7	potassium voltage-gated channel, subfamily H	−1.81	**−4.29**	−1.15
Kcns2	potassium voltage-gated channel, delayed-rectifier, subfamily S, member 2	−1.50	**−2.10**	−1.62
Scn4b	sodium channel, type IV, beta	**−2.05**	**−2.33**	**−2.65**
Slc16a6	solute carrier family 16 (monocarboxylic acid transporters), member 6	**−3.03**	**−3.20**	**−3.22**
Slc17a7	solute carrier family 17, member 7	−1.94	**−2.95**	−1.20
Slc1a5	solute carrier family 1 (neutral amino acid transporter), member 5	**−3.29**	−1.49	−1.85
Slc22a3	solute carrier family 22 (organic cation transporter), member 3	**−2.31**	**−3.59**	**−3.39**
Slc4a11	solute carrier family 4, sodium borate transporter, member 11	−1.87	**−2.71**	**−3.68**
Slc5a1	solute carrier family 5 (sodium/glucose cotransporter), member 1	−1.47	−2.07	**−2.57**
Slc9a3r1	solute carrier family 9 (sodium/hydrogen exchanger), member 3 regulator 1	**−1.92**	**−2.25**	**−2.21**
*Metabolic process*				
Stard4	StAR-related lipid transfer (START) domain containing 4	−1.37	−1.89	**−2.01**
Pla1a	phospholipase A1 member A	−2.07	**−2.77**	**−2.60**
Agpat7	acylglycerol-3-phosphate O-acyltransferase 7	−1.69	**−1.82**	**−1.85**
Asah3l	N-acylsphingosine amidohydrolase 3-like	−1.30	**−2.03**	**−1.22**
Hpse2	heparanase-2	−2.47	−2.35	**−3.27**
Mtmr1	myotubularin related protein 1	1.73	−1.42	**−5.07**
Neu2	neuraminidase 2	−1.74	**−2.06**	**−2.08**
*Neuropeptide/Hormone activity*				
Hcrtr1	hypocretin (orexin) receptor 1	−2.61	**−2.64**	−2.27
Nmu	neuromedin U	**−4.72**	**−7.99**	**−7.03**
Tshr	thyroid stimulating hormone receptor	−1.28	−2.19	**−3.45**
*Nucleotide synthesis*				
Atp8	ATP synthase F0 subunit 8	**−4.45**	−1.07	−1.62
Rrm2	ribonucleotide reductase M2	−1.45	−1.68	**−2.26**
*Protein binding*				
Admr	G protein-coupled receptor 182	**−3.16**	−1.06	−1.53
Cblb	Cbl proto-oncogene, E3 ubiquitin protein ligase B	−1.69	−1.66	**−2.03**
Fbf1	Fas (TNFRSF6) binding factor 1	−1.31	−1.53	**−1.97**
Fblim1	filamin binding LIM protein 1 (Fblim1)	**−4.50**	**−5.41**	**−3.55**
Hr	hairless	**−1.99**	**−1.94**	**−2.41**
Mtbp	Mdm2, p53 binding protein (mouse) binding protein	**−3.53**	**−3.53**	**−2.77**
Osbp2	oxysterol binding protein 2	−1.28	**−4.17**	−1.49
Pbk	PDZ binding kinase	−1.90	−3.75	**−5.36**
Pscdbp	pleckstrin homology, Sec7 and coiled-coil domains, binding protein	**−5.18**	**11.24**	**−26.12**
S100a3	S100 calcium binding protein A3	**−2.47**	**−2.35**	**−2.71**
S100a4	S100 calcium-binding protein A4	−1.88	**−1.82**	**−2.41**
Serinc2	serine incorporator 2	−2.87	−5.75	**−8.18**
Tnni3	troponin I type 3 (cardiac)	**−2.52**	**−3.21**	**−5.57**
Tpx2	TPX2, microtubule-associated, homolog	**−2.06**	**−3.81**	**−2.93**
Ttr	transthyretin	−8.88	**−9.37**	11.99
*Protein localization*				
Grik5	glutamate receptor, ionotropic, kainate 5	−2.58	**−2.13**	−2.03
Grin2c	glutamate receptor, ionotropic, NMDA2C	**−2.00**	**−2.01**	**−2.52**
Kdelr3	KDEL (Lys-Asp-Glu-Leu) endoplasmic reticulum protein retention receptor 3	−2.23	**−2.12**	−1.85
Wnk4	WNK lysine deficient protein kinase 4	−1.34	−1.66	**−1.82**
*Proteolysis*				
Ace	angiotensin I converting enzyme (peptidyl-dipeptidase A) 1	**−2.66**	**−2.90**	**−2.56**
Adamts19	ADAM metallopeptidase with thrombospondin type 1 motif, 19	−1.91	**−2.40**	**−4.19**
Asb2	ankyrin repeat and SOCS box-containing protein 2	−1.42	**−2.12**	**−2.53**
Dusp14	dual specificity phosphatase 14	−1.38	**12.14**	−3.78
Klk7	kallikrein-related peptidase 7	−4.54	**−5.31**	**−8.54**
Lct	lactase	−3.66	**−2.93**	**−3.80**
Mcpt4l1	mast cell protease 4-like 1provided	−1.74	−1.72	**−1.92**
Ppp1r1a	protein phosphatase 1, regulatory (inhibitor) subunit 1A	−1.65	**−2.60**	**−2.41**
Prss54	protease, serine, 54	**−4.24**	−1.68	−2.33
Sh3rf2	SH3 domain containing ring finger 2	−1.12	**−2.05**	−1.63
*Regulation of nucleotide*				
Rap1gap2	RAP1 GTPase activating protein 2	−1.56	−3.02	**−2.80**
*Sensory perception*				
Armc4	armadillo repeat containing 4	−1.10	−1.24	**−2.52**
Olr1260	olfactory receptor 1260	−2.79	−2.31	**−3.10**
Olr1579	olfactory receptor 1579	−3.05	−3.33	**−3.90**
Olr257	olfactory receptor 257	−1.01	**−2.29**	**−2.44**
Olr271	olfactory receptor 271	−2.55	−1.70	**−3.10**
Olr828	olfactory receptor 828	−2.15	**−2.21**	**−2.21**
Trpm8	transient receptor potential cation channel, subfamily M, member 8	−2.16	**−3.57**	−1.81
*Signal transduction*				
Arhgap25	Rho GTPase activating protein 25	**−3.30**	**−3.23**	**−4.25**
Arhgap9	Rho GTPase activating protein 9	−1.62	−2.18	**−2.44**
Arhgef19	Rho guanine nucleotide exchange factor (GEF) 19	−1.86	**−2.17**	−1.92
Bcar3	breast cancer anti-estrogen resistance 3	−1.77	**−2.26**	**−2.04**
Cacng1	calcium channel, voltage-dependent, gamma subunit 1	−2.88	**−5.20**	**−7.25**
Camk4	calcium/calmodulin-dependent protein kinase IV	−1.37	**−1.87**	**−1.92**
Cck	cholecystokinin	−1.68	**−3.43**	−1.96
Cnr1	cannabinoid receptor 1 (brain)	**−2.17**	**−2.44**	**−2.38**
Galnt14	UDP-N-acetyl-alpha-D-galactosamine:polypeptide N-acetylgalactosaminyltransferase 14	−1.98	**−2.41**	**−2.17**
Garnl4	RAP1 GTPase activating protein 2	−1.56	**−2.96**	**−2.80**
Gpcr12	G protein-coupled receptor 12	−1.55	−1.85	**−2.10**
Madh7	MAD homolog 7	−1.71	−2.35	**−1.97**
Mas1	MAS1 oncogene	−1.65	−2.69	−1.63
Mrgprb2	MAS-related G protein-coupled receptor, member X2-like	**−3.03**	−4.01	−3.45
Nrgn	neurogranin	−1.64	−1.93	**−1.87**
Nxph4	neurexophilin 4	−1.46	−1.81	**−2.74**
Rasd2	RASD family, member 2	−1.75	**−2.10**	**−2.09**
Rasgrp2	RAS guanyl releasing protein 2	−1.62	**−1.95**	**−2.29**
Rasl10a	RAS-like, family 10, member A	**−2.88**	**−2.57**	**−2.63**
Syt5	synaptotagmin V	−1.10	−1.31	**−1.92**
Tmem45b	transmembrane protein 45b	**−9.59**	**−8.29**	**−6.92**
Tmepai	transmembrane, prostate androgen induced RNA	−2.04	**−2.59**	**−2.27**
Vgf	VGF nerve growth factor inducible	−1.61	**−2.41**	**−2.37**
*Spindle organization*				
Aspm	asp (abnormal spindle) homolog, microcephaly associated	−1.68	−2.08	**−2.41**
Kntc2	kinetochore associated 2	−1.77	−1.72	**−3.10**
Ndc80	NDC80 homolog, kinetochore complex component	−2.41	−2.41	**−1.39**
Nusap1	nucleolar and spindle associated protein 1	−3.41	**−3.78**	**−3.78**
*Structural*				
Mospd4	motile sperm domain containing 4	−1.34	−1.58	**−2.12**
Tspear	thrombospondin-type laminin G domain and EAR repeats	−2.50	−1.81	**−4.08**
*Transcription*				
Ccdc77	coiled-coil domain containing 77	**−20.26**	−2.71	**−8.90**
Ccer1	Ccer1 – coiled-coil glutamate-rich protein 1	**−3.10**	−2.41	−1.39
Ccna2	cyclin A2	−2.00	**−5.94**	**−8.77**
Creb1	cAMP responsive element binding protein 1	−1.02	**−2.24**	1.06
Ebf1	early B-cell factor 1	−1.33	−1.77	−2.07
Fst	follistatin (Fst)	**−2.06**	**−2.73**	**−2.24**
Glrp1	glutamine repeat protein 1	−1.74	**−3.28**	−2.14
Hmgb2	high mobility group box 2	−1.48	**−2.07**	**−2.14**
Klf10	Kruppel-like factor 10	−1.31	**−2.88**	−1.79
Melk	maternal embryonic leucine zipper kinase	−1.98	**−2.00**	−1.90
Nanog	Nanog homeobox	−1.33	**−2.97**	−1.12
Nr4a2	nuclear receptor subfamily 4, group A, member 2	−1.20	**−2.80**	−1.21
Ns5atp9	NS5A (hepatitis C virus) transactivated protein 9	−1.89	**−4.35**	**−6.72**
Onecut2	one cut homeobox 2	−1.65	−1.95	**−2.25**
Pdlim1	PDZ and LIM domain 1	−2.14	**−2.62**	**−1.92**
Rarres1	retinoic acid receptor responder 1	**−2.24**	−1.72	−1.26
Rcor2	REST corepressor 2	−2.21	**−3.39**	−2.31
Rxrg	retinoid X receptor gamma	−1.63	**−1.84**	**−2.06**
Samd7	sterile alpha motif domain containing 7	**−3.19**	−1.61	−1.64
Sfmbt2	Scm-like with four mbt domains 2	−1.84	−2.07	**−2.91**
Tcf15	transcription factor 15	**−1.82**	**−1.98**	**−2.15**
Thrsp	thyroid hormone responsive protein	−1.60	**−2.20**	−1.63
Timeless	timeless circadian clock	−1.28	−1.68	**−1.95**
Traf4af1	TRAF4 associated factor 1	−2.53	**−8.01**	**−8.80**
Ttn	titin	−1.91	**−2.28**	**−3.76**
Ube2c	ubiquitin-conjugating enzyme E2C	−1.00	**−2.36**	**−2.42**
*Unknown*				
Linc00514	long intergenic non-protein coding RNA 514	**−2.14**	**−2.72**	**−3.34**
Lrrc10b	leucine rich repeat containing 10B	**−2.21**	**−3.29**	**−2.94**
Edc4	enhancer of mRNA decapping 4	**−2.40**	**−2.73**	**−2.73**

The animals were treated and microarray analyses were performed as described in the text. The number listed in bold under the representative columns (SMvSS, MSvSS, MMvSS) identify genes whose mRNA were significantly increased according to the following criteria: lesser than −1.8-fold, p<0.01. In some cases, values that are greater than −1.8-fold are not in bold because they did not reach the p value cut-off for the microarray analysis.

**Figure 4:**
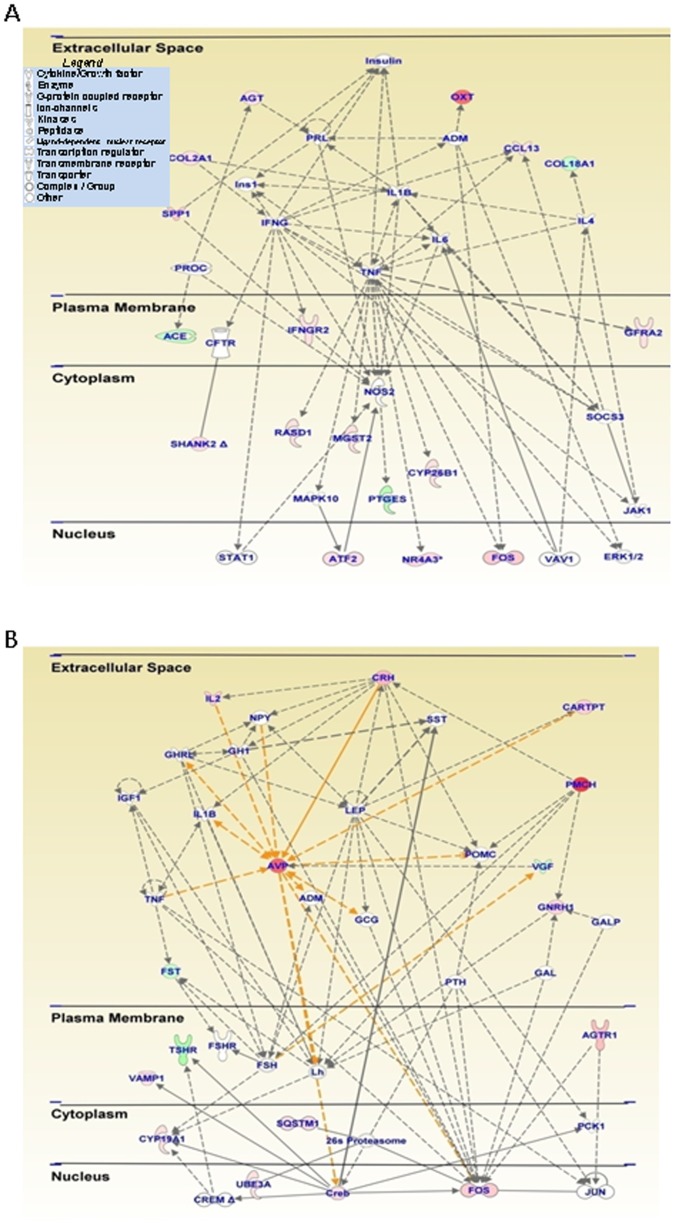
An acute METH injection produces differential gene expression in METH-pretreated rats. This network of related genes was generated as described in figure 2. Relationships between genes are also described in figure 2. The rats were injected with METH (10 mg/kg) and were euthanized 2 hours after a second injection of METH (2.5 mg/kg) given one month later. The genes were from the MMvSS comparison shown in figure 1. (**A**) This network shows genes that participate in cell signaling and molecular transport. (**B**) Acute METH injection influences the expression of neuropeptides in METH-pretreated rats. Several transcripts including Avp, Cart, and Crh (CRF) showed upregulation after the second METH injection. Note some of the similarities between this network and the one shown in figure 2.

The classes of genes that are differentially expressed in the MMvSS comparison are described in Table 2. That list includes *Avp* (15.99-fold), *Oxt* (14.91-fold), *Crh* (7.33-fold), and *Cart* (4.97-fold) that were upregulated. Several IEGs were also induced in that comparison. The genes affected in that group are involved in development and function of endocrine systems, cell signaling, and molecular transport. Figure 4A shows a network of genes involved in cell signaling and molecular transport whereas figure 4B shows a network that contains genes involved in development and endocrine functions. Canonical pathways affected by this treatment paradigm include cAMP-mediated signaling and CRH signaling. The potential role of some of these genes and pathways in METH-induced neuroadaptations is discussed below.

Table 4 shows some genes that were affected in the MMvMS comparison. These genes include *Npas4* (4.09-fold), *c-fos* (3.84-fold), *Nr4a3* (2.87-fold), *Arc* (2.09-fold), and *Crh* (1.83-fold) that were upregulated. These IEGs were also up-regulated in the SMvSS comparison but showed normal mRNA levels in the MS group. The fact that they are also significantly increased in the MMvSS and MMvMS comparison indicates that their responses to the second acute injection of METH were not significantly affected by the prior injection of the larger METH dose given one month earlier. This is in contrast to the observation for *Crh* that showed increased expression in the MS group, with a further potentiated response in the MMvMS comparison. Genes with altered gene expression participate in inflammatory responses, endocrine system disorders, cell signaling, and nervous system development. Canonical pathways included ERK/MAPK signaling and CRH signaling.

**Table 4:** Partial list of METH-upregulated genes in the MMvMS comparison.

Symbol	Definition	Fold changes
*Cell adhesion*		
Ptpn3	protein tyrosine phosphatase, non-receptor type 3	2.54
Ptpn4	protein tyrosine phosphatase, non-receptor type 4	2.39
*Cell migration*		
Cd34	CD34 antigen	5.27
Nck1	non-catalytic region of tyrosine kinase adaptor protein 1	2.77
Sorl1	sortilin-related receptor, L(DLR class) A repeats-containing	4.06
*Development*		
Col8a1	procollagen, type VIII, alpha 1	6.41
*Hormone activity*		
Crh	corticotropin releasing hormone	1.83
F5	coagulation factor 5	24.26
Igfbp2	insulin-like growth factor binding protein 2	2.16
Lhb	luteinizing hormone beta, transcript variant 2	1.82
Porf1	preoptic regulatory factor 1	1.94
Prok2	prokineticin 2	2.97
Scgb1c1	secretoglobin, family 1C, member 1	6.95
Sostdc1	sclerostin domain containing 1	30.53
Ttr	transthyretin	112.39
*Immune response*		
Bpil1	bactericidal/permeability-increasing protein-like 1	3.12
Nfil3	nuclear factor, interleukin 3 regulated	2.01
*Ion binding*		
Arid5a	AT rich interactive domain 5A	7.56
*Ion transport*		
Kcnh7	potassium voltage-gated channel, subfamily H	3.75
Kcnt2	potassium channel, subfamily T, member 2	2.11
Slco1a5	solute carrier organic anion transporter family, member 1a5	4.00
Steap1	six transmembrane epithelial antigen of the prostate 1	6.18
*Metabolic process*		
Asah2	N-acylsphingosine amidohydrolase 2	3.54
Glb1l3	galactosidase, beta 1-like 3	3.03
Mtmr1	myotubularin related protein 1	4.40
*Protein binding*		
Eif3s12	eukaryotic translation initiation factor 3, subunit 12	2.00
Kpna4	karyopherin (importin) alpha 4	1.82
*Protein transport*		
Nup133	nucleoporin 133	2.33
*Proteolysis*		
Dusp1	dual specificity phosphatase 1	1.88
Dusp4	dual specificity phosphatase 4	3.80
Ppp1r15b	protein phosphatase 1, regulatory subunit 15b	1.99
Usp31	ubiquitin specific protease 31	2.91
*Sensory perception*		
Mfrp	membrane frizzled-related protein, transcript variant 1	14.56
*Signal transduction*		
Adcy6	adenylate cyclase 6	3.28
Arc	activity regulated cytoskeletal-associated protein	2.09
Lhfpl4	lipoma HMGIC fusion partner-like protein 4	2.66
Manba	mannosidase, beta A, lysosomal	2.39
Pank3	pantothenate kinase 3	1.85
Plac9	placenta-specific 9	4.06
Rab33b	RAB33B, member of RAS oncogene family	4.18
*Transcription*		
Bmp7	bone morphogenetic protein 7	1.81
Egr4	early growth response 4	2.05
Fos	FBJ murine osteosarcoma viral oncogene homolog	3.84
Msc	musculin	6.35
Npas4	neuronal PAS domain protein 4	4.09
Nr1i3	nuclear receptor subfamily 1, group I, member 3	2.61
Nr2c2	nuclear receptor subfamily 2, group C, member 2	2.78
Nr4a2	nuclear receptor subfamily 4, group A, member 2	2.32
Nr4a3	nuclear receptor subfamily 4, group A, member 3	2.87

The animals were treated and microarray analyses were performed as described in the text. The gene list was generated based on the following criteria: greater than +1.8-fold, p<0.01. The genes within each class are listed in descending order based on fold-changes in MMvMS comparison.

### Quantitative PCR analysis

**Figure 5:**
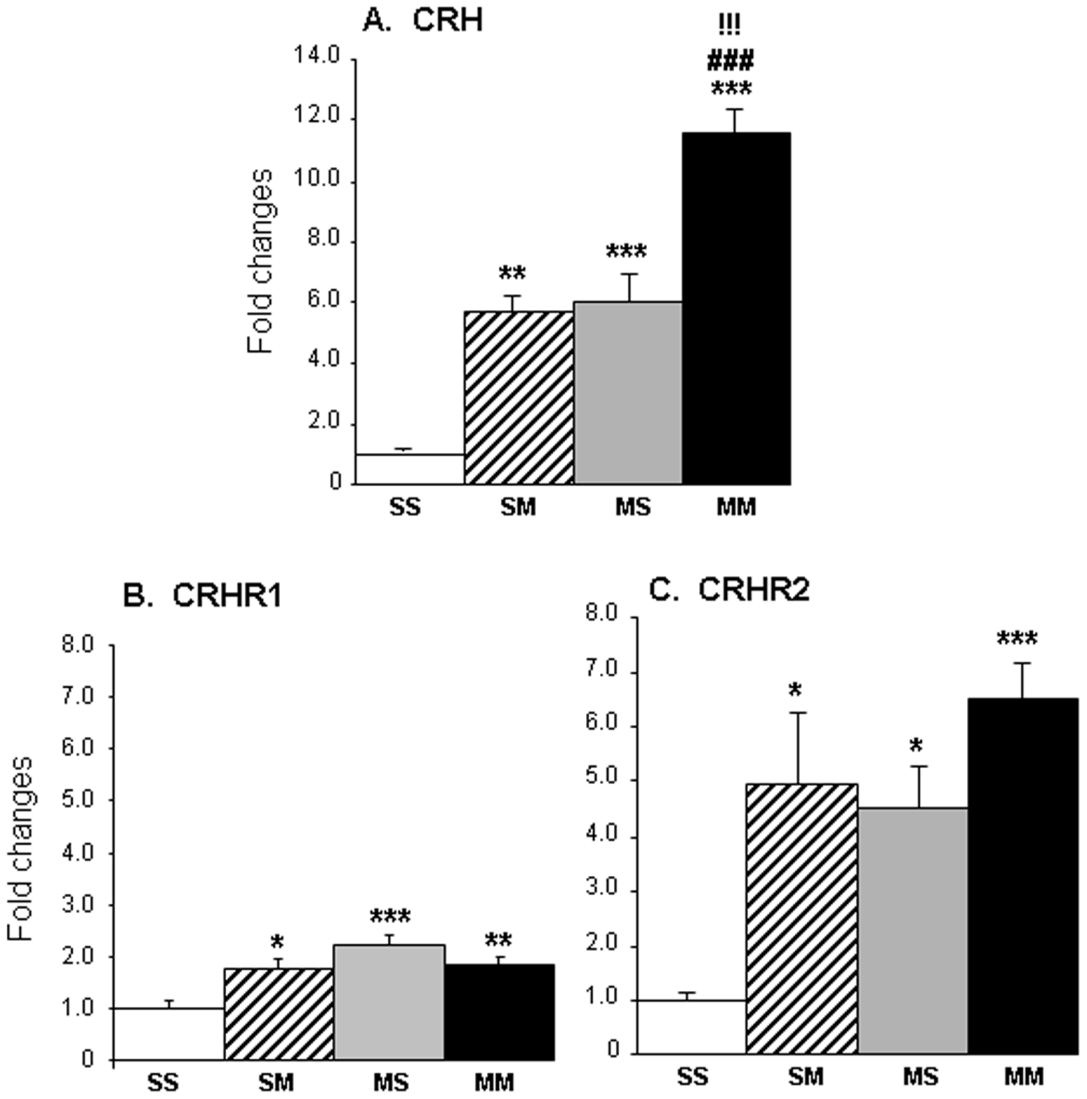
METH induced changes in the expression of neuropeptides in the rat NAc. The figure shows the acute and more delayed effects of METH injections on the mRNA levels of (**A**) CRH, (**B**) CRHR1, and (**C**) CRHR2. The PCR data confirmed the changes in expression in CRH expression observed in the microarray data and document changes in CRH receptor expression. Rats were injected (4 rats in SS; 6 rats in SM; 7 rats in each MS and MM groups respectively) and total RNA was extracted from the NAc as described in the text. Statistical significance was determined by ANOVA followed by post-hoc tests. The null hypothesis was rejected at *P*<0.05. Key to statistics: * = *P*<0.05, ** = *P*<0.01; *** = *P*<0.001, in comparison to the SS group; ### = *P*<0.001, in comparison to the SM group; !!! = *P*<0.001, in comparison to the MS group.

We used qPCR to confirm the changes in gene expression of some genes of interest. Figure 4 shows METH-induced changes in mRNA levels for *Crh* and its receptors (4 rats in SS; 6 rats in SM; 7 rats in each MS and MM groups respectively). A single injection of METH (2.5 mg/kg) caused significant increases (5.7-fold) in *Crh* mRNA levels (Fig. 5A) in saline-pretreated rats (SMvSS). Similar increases (6.0-fold) were observed in rats that had been injected with an injection of METH (10 mg/kg) a month earlier (MSvSS). As per the array data, the injection of the smaller METH dose caused further increases (11.6-fold) in *Crh* expression in rats pretreated with METH (10 mg/kg dose) a month earlier, with increases in the MMvSS comparison being significantly higher than in the SMvSS and MSvSS comparisons (Fig. 5A). We also measured the expression of CRH receptors after the METH injections. Acute METH caused significant increases *Crhr1* mRNA levels in saline-pretreated (1.8-fold, SMvSS) and in METH-pretreated (1.8-fold, MMvSS) rats (Fig. 5B). There were also increases (2.2-fold) in the animals that had received the larger METH dose a month earlier (MSvSS, Fig. 5B). Interestingly, we observed significant greater increases in *Crhr2* mRNA expression in the SMvSS (∼5-fold), MSvSS (4.5-fold), and MMvSS (6.5-fold) comparisons (Fig. 5C) than those observed for *Crhr1* expression (compare Fig. 5B to 5C).

**Figure 6:**
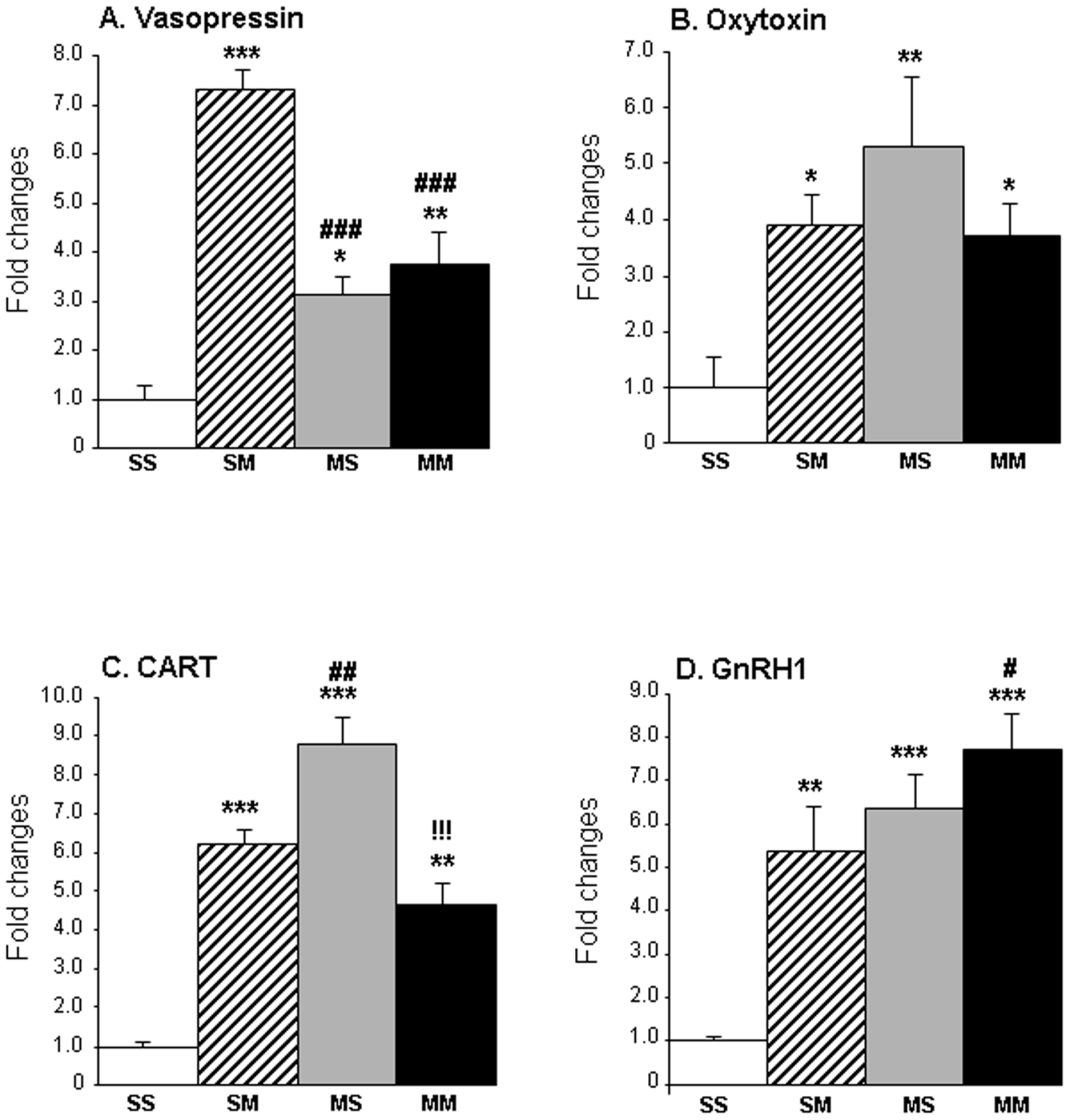
METH induced changes in the expression of neuropeptides in the rat NAc. The figure shows the acute and more delayed effects of METH injections on the mRNA levels of (**A**) Vasopressin, (**B**) Oxytocin, (**C**) CART, and (**D**) GnRH1 measured by quantitative PCR. The PCR data confirmed the microarray data. Key to statistics: * = *P*<0.05, ** = *P*<0.01; *** = *P*<0.001, in comparison to the SS group; # = *P*<0.05; ## = *P*<0.01; ### = *P*<0.001, in comparison to the SM group; !!! = *P*<0.001, in comparison to the MS group.

**Figure 7:**
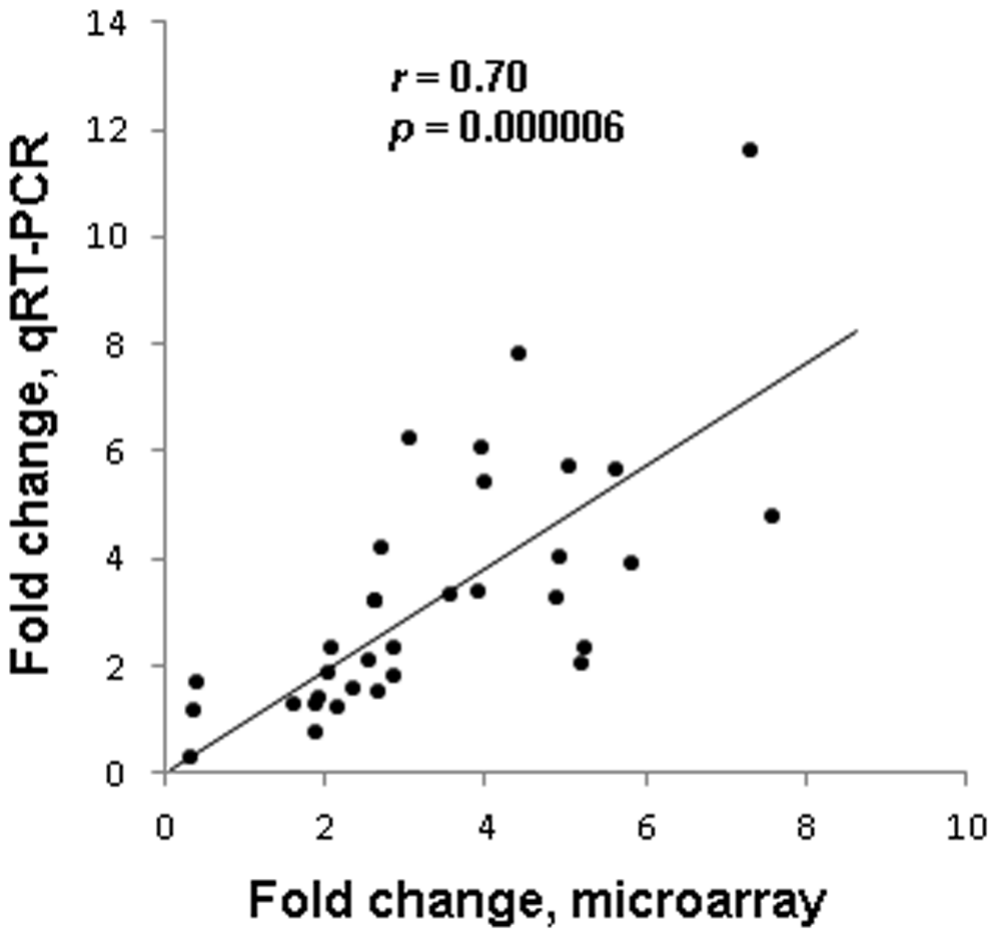
qPCR validation of METH-induced changes identified by microarray analysis. There is a significant correlation between METH-induced changes in the expression of genes identified by microarray analysis and validated by qRT-PCR.

Figure 6A shows that there were increased Avp mRNA expression in the SMvSS (7.2-fold), the MSvSS (3-fold), and MMvSS (3.5-fold) comparisons. METH injections also caused increased oxytocin expression in SMvSS (3.9-fold), MSvSS (5.3-fold) and MMvSS (3.7-fold) (Fig. 6B). The single METH (10 mg/kg), given one month earlier caused greater changes in *Cart* expression in the MSvSS (8.8-fold) comparison than those observed in the SMvSS (6.2-fold) and MMvSS (4.7-fold) (Fig. 6C) comparisons. Figure 6D shows the effects of METH on *Gnrh1* expression. As per the array data, acute METH increased GnRH1 in the SMvSS (5.4-fold) and MMvSS (7.7-fold) comparisons. The single injection of the larger METH dose also caused long-lasting changes in *Gnrh1* expression in the MS (6.3-fold) group. The changes in *Gnrh1* expression in the MMvSS comparison were significantly higher than those observed in the SMvSS, suggesting that the previous METH injection had enhanced the effects of the second METH injection. Importantly, a correlation analysis revealed a significant positive correlation (*r* = 0.70, *p* = 0.000006) between the microarray and qPCR data (Fig. 7).

## Discussion

The present study shows that an acute METH (2.5 mg/kg) injection can cause differential changes in gene expression in the rat NAc. The transcriptional profiles observed after the METH injections indicate that METH can induce the expression of several genes that participate in the control of transcription and cAMP signaling in the NAc. These findings are consistent with the fact that METH exerts its effects, in part, by releasing DA followed by stimulation of DA D1 receptors that are linked to cAMP activation [6], [7]. In fact, use of the DA antagonist, SCH23390, produces substantial inhibition of the acute transcriptional effects of METH [6], [7]. We also show, for the first time, that a moderate dose of METH (10 mg/kg) can have long-lasting effects on gene expression in the NAc. Pre-exposure to this METH dose influenced METH-induced changes in the expression of many genes upon re-exposure of the rats to a smaller METH dose given a month later. For example, prior exposure to METH led to the potentiation of increased *Crh* mRNA expression induced by a second METH injection. Taken together, these METH-induced alterations in gene expression in the NAc support the notion that METH can exert pleiotropic effects in the brain [4], [6]–[8]. This conclusion is consistent with results of the pathway analyses that identified METH-induced changes in canonical pathways related to cell-to-cell communication, endocrine functions, and inflammatory responses. The identification of these pathways is of interest and adds to the literature that suggests addiction to psychostimulant involves perturbations in synaptic pathology in a number of neurotransmitter systems [35], resulting, in part, from drug-induced transcriptional changes in the brain [37], [38].

Although drug-induced sensitization has been studied mostly in models where repeated injections of psychostimulant are given [14], considerable evidence exists to suggest that a single injection of drugs of abuse can cause long-term neurochemical, neuroendocrine, and physiological effects in rodents. For example, enhanced amphetamine-induced striatal DA release was reported after a single injection of either amphetamine [18] or cocaine [17]. A single cocaine injection enhanced NAc DA release in response to a second cocaine injection given a week later [16]. A single in vivo exposure to cocaine can cause prolonged long-term potentiation in NAc neurons [34] [28]. A single higher dose of cocaine (20 mg/kg) also enhanced the response to a second dose of cocaine (10 mg/kg) and enhanced the expression *GluR1* and *GAP-43* mRNA in the NAc [39]. Similar observations have been reported in response to a two-injection pattern of amphetamine administration [19] in a paper that uses a paradigm similar to the METH injection schedule used in the present study. These authors reported enhanced biochemical effects of a second amphetamine (1 mg/kg) injection after prior exposure to the drug (5 mg/kg) [19]. Together, these reports are consistent with our finding that the prior injection of a moderate dose of METH (10 mg/kg) caused enhancement of the increased *Crh* mRNA expression induced by the administration of a lower METH dose (2.5 mg/kg). CRH/CRF is a 41 amino acid peptide that was identified as a hypothalamic releasing factor that stimulated the secretion of adrenocorticotropic hormone (ACTH) and beta-endorphin [40], and of corticosterone [41]. Subsequent studies also demonstrated that CRH [42]–[44] and CRH receptor proteins and mRNAs [45]–[48] are widely distributed in the central nervous system (CNS).

These observations suggest that CRH and its receptors might serve to integrate physiological responses to stressful stimuli [49]–[51]. Our findings that METH can cause increased mRNA expression of *Crh* and of its receptors (*Crfr1/Crhr1 and Crfr2/Crhr2*) are consistent with reports that both *Crh* and *Crhr1* mRNA levels are increased by stress [52]–[54] and by intracerebral administration of CRH itself [55], [56]. Our results are also consistent with previous data that had implicated the CRH system in METH-induced locomotor effects [57], [58]. Our data are also in line with the reported activation of the hypothalamic-pituitary-adrenal (HPA) axis by various drugs of abuse [59]. Our observations of METH-induced *Crhr1* and *Crhr2* expression are also consistent with a previous report that *Crhr1* gene expression in limbic brain regions is important to neuroendocrine responses to stress [60] and with the fact that *Crhr2*-mutant mice are more sensitive to stress [61]. Interestingly, we found that METH caused greater increases in *Crhr2* than in *Crhr1* expression in all three groups of METH-treated rats. These observations are consistent with those of other investigators who had reported differential responses in *Crhr1* and *Crhr2* expression associated with stress, with CRHR1 being internalized and CRHR2 being recruited to the cell membrane [62], [63]. They are also consistent with reports that chronic cocaine facilitates the electrophysiological effects of CRHR2 stimulation [64], [65]. Importantly, our observations of METH-induced increased *Crh* and *Crhr* mRNA expression are in agreement with previous results that a single injection of amphetamine (1 mg/kg) induced time-dependent sensitization of the HPA axis such that, by 1–3 weeks after a prior injection of amphetamine (5 mg/kg), there was an augmented secretion of ACTH and corticosterone consequent to a second injection of amphetamine (1 mg/kg) [19]. When taken together with previous findings, the present observations add more support to the accumulating evidence that disturbances in CNS stress response systems might play an important role in long-term molecular adaptations consequent to psychostimulant exposure [27]. Our findings might also be relevant to stress- and drug-induced reinstatement of drug taking, with differential involvement of various limbs of these neuroendocrine cascades being more or less involved in certain aspects of addictive behaviors [66], [67]. Together with our observations of METH-induced greater changes in *Crhr2* than in *Crhr1* expression, the reviewed literature implicates both CRH receptors in the behavioral and physiological effects of psychostimulant, with CRHR2 playing a more prominent role that it had been assigned so far [68].

We found that the METH (10 mg/kg) injection caused long-lasting increases in *Avp* mRNA levels. Arginine vasopressin (AVP), one of the first identified neuropeptides, is found predominantly in the hypothalamus [69]. AVP is located in other brain regions including the NAc [70] where it interacts with specific AVP receptors [71], [72]. AVP also interacts with the HPA axis, the extended amygdala, and monoaminergic systems [73]. AVP is co-regulated with CRH [53] and AVP-expressing hypothalamic neurons co-express CRHR1 and CRHR2 receptors [74], results that support the co-involvement of CRH and AVP in stress responses [75], [76]. This discussion is consistent with the pathway analysis that shows a direct interaction of AVP and CRH (Fig. 2). Interestingly, the IPA also shows that both peptides are linked to the expression of the IEGs, *c-fos* and *Creb*, whose mRNAs are also induced by the acute METH injection (Fig. 2). Moreover, our findings of increased *Avp* mRNA expression are consistent with reports that administration of another stimulant, cocaine, can increase *Avp* mRNA in the NAc [70]. *Avp* mRNA is increased within 3 hours after cessation of chronic cocaine administration (3×15 mg/kg per day for 14 days) [77] and the increased mRNA expression persists for several weeks during protracted withdrawal from escalating-dose cocaine administration [78]. In addition to the effects of cocaine, stimulation of the mesolimbic system by local injection of a substance P analog into the ventral tegmental area also induces AVP release [79], [80], indicating that this neuropeptide might indeed be an important mediator of some of the physiological effects of psychostimulant since these drugs exert their varied effects through stimulation of monoaminergic systems [1]–[3]. This suggestion is consistent with the demonstration that AVP and its analogues can reduce cocaine self-administration in rats [81], [82]. Taken together with cocaine administration results, our observation of METH-induced prolonged increases in *Avp* mRNA levels supports the notion that AVP might also participate in neuroadaptive responses triggered by repeated exposure to psychostimulants.

CART is another peptide whose mRNA expression was induced in the different groups of METH-injected rats. CART is a neuropeptide that was discovered using PCR differential display as a rat brain mRNA that responded to cocaine and amphetamine [83], [84]. CART is distributed throughout the brain [84] and is thought to be relevant to the effects of psychostimulants on the reward system in the brain [36], [85]–[90]. CART also participates in stress responses [91], [92]. Of considerable interest to our present observations is the report that CART can activate the HPA axis through a CRH receptor-dependent mechanism [93]. Thus, together with the observed changes in *Crh* and *Avp* mRNA expression after the METH injections, the METH-induced increased *Cart* mRNA expression suggests that METH can cause coordinated changes in the expression of neuropeptides that modulate stress responses in the brain. Similar observations have been made in response to other stressful events [94], [95].

The acute and prolonged changes in *Oxt* expression caused by METH are also of singular interest. Oxytocin (Oxt) is a nanopeptide [96] that is involved in affiliative [97], grooming [98], maternal [99], pair bonding [100], and other complex behaviors [101]–[104]. Our observations of METH-induced changes in *Oxt* expression are consistent with those of several studies that have now reported the potential involvement of Oxt in the behavioral and biochemical effects of psychostimulants [105], [106]. For example, it has been reported that Oxt is itself rewarding when tested in the conditioned place preference (CPP) paradigm [107]. In contrast, Oxt reduced cocaine-induced hyperactivity [108], stereotypy [109], and self-administration [110]. More recent studies have provided evidence that Oxt can decrease METH-induced hyperactivity [111], CPP [112], [113], METH self-administration [114], and relapse to METH-seeking behaviors [114], [115]. Oxt also decreased METH-induced activation of the subthalamus nucleus and of the NAc core [116]. Although not yet completely elucidated, these effects of Oxt on METH-induced behaviors are probably due, in part, to its effects on the dopaminergic systems since Oxt can reduce METH-induced DA release in the NAc [111] and serve as a neuromodulator of dopaminergic functions in various behavioral models [117], [118]. When taken together with our present findings, the reviewed literature supports the idea that more studies of this important neuromodulatory system are warranted in models of drug addiction [106]. In view of the observed effects of METH on both Oxt and Avp expression, it will be important to dissect the specific role of each peptide in drug addiction because they are both involved in the modulation of various mammalian behaviors [99], [102], [119].

In summary, this is the first demonstration that a single injection of a moderate METH dose can cause long-lasting alterations in gene expression in the NAc. These changes include prolonged overexpression of mRNAs that code for several neuropeptides including AVP, CART CRH, CART, and OXT that are involved in multipronged neuroendocrine responses to environmental stimuli stress and affiliative interactions. The augmented responses in CRH transcript expression suggest that the peptide might also play important roles in the molecular events that drive plastic alterations in the NAc in response to METH exposure, in a fashion consistent with stress-induced dynamic changes in the brain [51]. More studies are needed to further dissect the role of these neuropeptides in molecular neuroadaptations that are consequent to repeated drug exposure. The impact of these changes within specific cell types within the NAc core and shell subregions will also need to be elucidated.

## Materials and Methods

### Animals, drug treatment, and tissue collection

Male Sprague-Dawley rats (Charles River Labs, Raleigh, NC, USA), weighing 375±25 g, were used in the experiments. Rats were housed in a temperature-controlled (22.2+0.2°C) room with free access to food and water. All animals were allowed to acclimate to the facility for one week. At first, the animals received a single injection of either saline or METH (10 mg/kg). This injection was followed after a month delay by a second injection of either saline or METH (2.5 mg/kg). This pattern of injections yielded four groups of rats: saline-pretreated and saline-challenged (SS); saline-pretreated and METH-challenged (SM); METH-pretreated and saline-challenged (MS) and METH-pretreated and METH-challenged (MM), summarized in Figure S1 in File S1. Proper handling techniques were used to reduce stress to the animals during injections. Rats were euthanized at 2 hours after the second injection of either METH or saline. NAc tissues were dissected and immediately frozen on dry ice. The pattern of using a larger dose of METH followed by a second lower dose is consistent with studies in which single doses of either cocaine [120] or amphetamine [19] have been used to investigate biochemical sensitization to a second lower dose. Similarly, lower challenge doses of psychostimulants are used when measuring biochemical sensitization after repeated intermittent injections of increasing doses of either cocaine or amphetamine (see [121], [122] and references therein).

Initially, the brain was placed on its dorsal surface on a metal plate that was kept cold on crushed ice. The nucleus accumbens (containing both core+shell subregions) was dissected from the ventral surface of the brain. A wedge of brain tissue is obtained by cutting along two lines: one extending from the base of the lateral ventricle, through the anterior commissure to the medial edge of the lateral olfactory tract and the other connecting the base of the lateral ventricle and the base of the brain.

The tissues were kept at −70°C until they were processed for HPLC analysis or RNA extraction. All animal use procedures were according to the NIH Guide for the Care and Use of Laboratory Animals and were approved by the National Institute of Drug Abuse-/Intramural Research Program (IRP) Animal Care and Use Committee (NIDA/IRP-ACUC).

### HPLC Analysis

Monoamine levels in the NAc were quantified by HPLC with electrochemical detection as described in our previous publications [5].

Briefly, NAc was homogenized in 0.01 M HClO_4_ and centrifuged at 14, 000×g for 15 min. DA, DOPAC, HVA, 5-HT, and 5-HIAA levels were measured by HPLC with electrochemical detection. The analytical column was SunFire C18 (5 µm particle size, 4.6×150.0 mm) from Waters (Waters Corp., Millford, MA). The mobile phase was 0.01 M sodium dihydrogenphosphate, 0.01 M citric acid, 2 mM sodium EDTA, 1 mM sodium octylsulfate, 10% methanol, pH 3.5 at flow rate 1.0 ml/min and temperature 35°C. The installation consisted of Waters 1525 Binary HPLC pump and Esa Coulochem III electrochemical detector (Thermo Fisher Scientific, Sunnyvale, CA). The glassy carbon electrode was used at a potential of 0.75 V. Peak areas and sample concentrations were calculated with the proprietary software program, Breezes (Waters Corp.). The program was used to calculate peak height and to integrate known standards for the HPLC data. Contents of DA, DOPAC and HVA were calculated as pg/mg of tissue weight.

### RNA extraction, microarray hybridization, and data analysis

Total RNA was isolated according to the manufacturer's manual using Qiagen RNeasy mini kit (Qiagen, Valencia, CA, USA). RNA integrity was detected using an Agilent 2100 Bioanalyzer (Agilent Technologies, Santa Clara, CA, USA) and showed no degradation (see Table S1 in File S1 for details). Microarray hybridization was carried out using RatRef-12 Expression BeadChips arrays (22,523 probes) (Illumina Inc., San Diego, CA) essentially as previously described by our laboratory [5], [11]. Briefly, a 600 ng aliquot of total RNA from each NAc sample was amplified using Illumina RNA Amplification kit (Ambion, Austin, TX). Single-stranded RNA (cRNA) was generated and labeled by incorporating biotin-16-UTP (Roche Diagnostics, Indianapolis, IN). 750 ng of each cRNA sample were hybridized to Illumina arrays at 55°C overnight according to the Whole-Genome Gene Expression Protocol for BeadStation (Illumina Inc.). Hybridized biotinylated cRNA was detected with Cyanine3-streptavidin (GE Healthcare, Piscataway, NJ) and quantified using Illumina's BeadStation 500GX Genetic Analysis Systems scanner. The Illumina BeadStudio software was used to measure fluorescent hybridization signals and to subtract the background signal. Background subtracted data was imported into GeneSpring software v.12 (Agilent Technologies) and baseline normalization to the median values of each array (n = 24) were performed. The normalized data were used to identify changes in gene expression after the injection of METH. A gene was identified as significantly changed if it showed increased or decreased expression according to an arbitrary cut-off of 1.8-fold changes at *P*<0.01 using unpaired t-test in the GeneSpring statistical package. Similar criteria have been used successfully in our other studies [5], [11]. The results are reported as fold changes calculated as the ratios of normalized gene expression data for METH-treated groups (SM, MS, and MM) in comparison to the control group (SS).

### Quantitative polymerase chain reaction (qPCR)

A portion of the total RNA (0.5 µg) isolated from the NAc samples used in the microarray analysis (Table S1 in File S1) was reverse-transcribed with oligo dT primers using Advantage RT-for-PCR kit (Clontech, Mountain View, CA). qRT-PCR was performed as described previously [5], [11] with Roche LightCycler 480 II (Roche Diagnostics Corp., Indianapolis, IN) using iQ SYBR Green supermix (Bio-Rad, Hercules, CA). For all qRT-PCR experiments, individual data were normalized using the corresponding OAZ1 (ornithine decarboxylase antizyme 1) mRNA level. OAZ1 was used because its expression did not show any significant changes at any time points after the METH injection. The results are reported as fold changes calculated as the ratios of normalized gene expression data for METH-treated groups (SM, MS, and MM) in comparison to the control group (SS). The primers for RT-PCR were synthesized at the Synthesis and Sequencing Facility of Johns Hopkins University (Baltimore, MD, see Table S2 in File S1).

### Statistical Analyses

All data are presented as means ± SEM. Statistical analyses were performed using one-way ANOVA analysis followed by Fisher's protected least significant difference (StatView 4.02, SAS Institute, Cary, NC). The null hypothesis was rejected at p≤0.05.

## Supporting Information

File S1 **Figure S1, Pictogram showing the drug treatment schedule.Table S1, RNA Integrity Number (RIN) of Samples. Table S2, List of RT-PCR primers.**
(DOCX)
