# Supplementary material for: Enhanced Upregulation of CRH mRNA Expression in the Nucleus Accumbens of Male Rats after a Second Injection of Methamphetamine Given Thirty Days Later
Source: PLoS One. 2014 Jan 27;9(1):e84665. doi: 10.1371/journal.pone.0084665 (PMC3903495; doi:10.1371/journal.pone.0084665)
Supplement: File S1 — Figure S1, Pictogram showing the drug treatment schedule.Table S1, RNA Integrity Number (RIN) of Samples. Table S2, List of RT-PCR primers. (DOCX) [file pone.0084665.s001.docx]

| **Table S1: RNA Integrity Number (RIN) of Samples** | | | |
| --- | --- | --- | --- |
| **Sample** | **RIN** | **Used in Microarray** | **Used in RT-PCR** |
| SS 1 | 8.2 | + | + |
| SS 2 | 8.2 | + | + |
| SS 3 | 8.2 | + | + |
| SS 4 | 8.9 | + | + |
| SM 1 | 8.1 | + | + |
| SM 2 | 8.5 | + | + |
| SM 3 | 8.2 | + | + |
| SM 4 | 8.3 | + | + |
| SM 5 | 8.6 | + | + |
| SM 6 | 8.6 | + | + |
| MS 1 | 8.9 | + | + |
| MS 2 | 8.5 | + | + |
| MS 3 | 9.1 | + | + |
| MS 4 | 9.1 | + | + |
| MS 5 | 7.7 | + | + |
| MS 6 | 8.1 | + | + |
| MS 7 | 8.4 | + | + |
| MM 1 | 8.4 | + | + |
| MM 2 | 8.3 | + | + |
| MM 3 | 8.3 | + | + |
| MM 4 | 9.2 | + | + |
| MM 5 | 8.9 | + | + |
| MM 6 | 8.6 | + | + |
| MM 7 | 8.5 | + | + |
| RIN takes into account the values for 28S, 18S and small ribosomal RNAs compared to one another as well as the total signal. RIN is used for assigning integrity values to RNA measurements. The higher RIN correlates with better outcomes of RT-PCR and microarrays. Schroeder et. al. (2006) demonstrated that the RIN greater than 6 to be of high quality. Note, our samples' RIN is greater than 7.7. | | | |
|  | | | |

| **Table S2: List of RT-PCR primers** | |  |
| --- | --- | --- |
|  |  |  |
| **Primer Name** | **Forward Sequence** | **Reverse Sequence** |
| Avp | GAG TGT CGA GAG GGT TT | GGC GAT GGC TCA GTA GA |
| Cart | TGG GAA GAA GAG GGA CT | TAA TTT GCA CAT GCT TCC A |
| Cck | AAC AAC CAC GCA CAC GA | CCA GAG GGA AAC ATT GCC |
| Crf | CCT CGC AGA ACA ACA GTG | CCT CAG AAG GTG GAA GGT G |
| Crfr1 | GTC CCT GAC CAG CAA TGT TT | CGG AGT TTG GTC ATG AGG AT |
| Crfr2 | TCA TTG GAT GGT GCA TAC | TTG ATG AGG AGC ACG AG |
| Gnrh1 | GGA AGA GAA ATA CTG AAC ACT TGG T | AGA TCC CTA AGA GGT GAA CGG |
| Oxt | GAT ATG CGC AAG TGT CT | GAG GGC AGG TAG TTC TC |
| Sst | CTT CTT CTG GAA GAC ATT CAC | ATT TAC AGT CTT CAA TTT CTA ACG |
| OAZ1 | GCT CAC TCC ATT AGC GG | CCA GAC TTC AAA GGA GG |

**Figure S1**

**Pictogram showing the drug treatment schedule.** The rats received an initial injection of either saline or METH (10 mg/kg). This injection was followed after an interval of 30 days by a challenge injection of either saline or METH (2.5 mg/kg). This pattern of injections yielded four groups of rats: saline-pretreated and saline-challenged (SS); saline-pretreated and METH-challenged (SM); METH-pretreated and saline-challenged and METH-pretreated and METH-challenged (MM).
